# Canadian physicians’ responses to cross border health care

**DOI:** 10.1186/1744-8603-10-20

**Published:** 2014-04-03

**Authors:** Vivien Runnels, Ronald Labonté, Corinne Packer, Sabrina Chaudhry, Owen Adams, Jeff Blackmer

**Affiliations:** 1 Institute of Population Health, University of Ottawa, Ottawa, ON, Canada; 2 University of Saskatchewan, Saskatoon, Saskatchewan, Canada; 3 Health Policy and Research Department, Canadian Medical Association, Ottawa, ON, Canada; 4 Office of Ethics, Canadian Medical Association, Ottawa, ON, Canada

**Keywords:** Medical tourism, Out-of-country care, Cross-border care, International health care, Survey

## Abstract

**Background:**

The idea for this survey emanated from desk research and two meetings for researchers that discussed medical tourism and out-of-country health care, which were convened by some of the authors of this article (VR, CP and RL).

**Methods:**

A *Cross Border Health Care Survey* was drafted by a number of the authors and administered to Canadian physicians via the Canadian Medical Association’s e-panel. The purpose of the survey was to gain an understanding of physicians’ experiences with and views of their patients acquiring health care out of country, either as medical tourists (paying out-of-pocket for their care) or out-of-country care patients funded by provincial/territorial public health insurance plans. Quantitative and qualitative results of the survey were analyzed.

**Results:**

631 physicians responded to the survey. Diagnostic procedures were the top-ranked procedure for patients either as out-of-country care recipients or medical tourists. Respondents reported that the main reason why patients sought care abroad was because waiting times in Canada were too long. Some respondents were frustrated with a lack of information about out-of-country procedures upon their patients’ return to Canada. The majority of physician respondents agreed that it was their responsibility to provide follow-up care to medical travellers on return to Canada, although a substantial minority disagreed that they had such a responsibility.

**Conclusions:**

Cross-border health care, whether government-sanctioned (out-of-country-care) or patient-initiated (medical tourism), is increasing in Canada. Such flows are thought likely to increase with aging populations. Government-sanctioned outbound flows are less problematic than patient-initiated flows but are constrained by low approval rates, which may increase patient initiation. Lack of information and post-return complications pose the greatest concern to Canadian physicians. Further research on both types of flows (government-sanctioned and patient-initiated), and how they affect the Canadian health system, can contribute to a more informed debate about the role of cross-border health care in the future, and how it might be organized and regulated.

## Background

Canada, like many countries, is experiencing an increase in the number of patients who seek health care outside of its borders. Canadians who do so generally fall into two categories. If they require health care that is not available close to home or in their own province or territory they may be able to access health care in another province or out of country (often the USA) through a system of prior approval that is organized and administered by provincial/territorial ministries of health. Referred to as “out-of-country care” (OOCC), this is funded by provincial/territorial public health insurance plans and usually involves insured procedures for which there is a delay which poses catastrophic health risks to the patient [1]. Medical “tourism” (MT), the second category, is planned and organized by the individual without the involvement of public health insurance plans [2]. (We put “tourism” in quotes since, despite the adoption of this term, the primary reason for such travel is medical treatment, not tourism). MT “refers to cross-border health care motivated by lower cost, avoidance of long wait times, or services not available in one’s own country” [2], with the patient funding his/her health care out-of-pocket. MT travel for the purpose of health care results from a patient’s personal choice and decision to seek medical care abroad in the absence of a physician’s formal referral for care or approval from public health insurance plans [3,4].

Between 2004 and 2009, the annual growth rate of imports of health care services (referred to as imports because they are purchases of medical services and goods made by resident Canadians while they are abroad) was 1% [5]. Imports of health care services as share of total health expenditure in 2009 stood at 0.24%. The figures only provide a rough guide to growth rates in medical tourism. However, as a highly publicized component of trade in health services in which Canadians have an interest as demonstrated through media reports, an apparent growth in the medical tourism industry’s infrastructure, and a variety of list servs, MT appears to be on the rise [6]. This rise reflects, in part, the globalization of health care, in which (generally) private health care providers seek to increase their revenues, maintain bed-occupancy rates and/or support development of high-technology medicine by attracting fee-paying (out of pocket, privately or publicly insured) international patients [1]. Many low- and middle-income countries are engaged in promoting MT, often with the direct support of their governments which regard health care exports as a means of generating foreign currency earnings. That many of these countries have poor health care access for their own citizens raises important questions of equity and ethics, beyond the scope of this article but part of a separate study with which some of the authors are associated (RL, VR, CP). Our focus in this article is how Canadian physicians, who are responsible for the care of Canadian patients, engage in or otherwise feel about this perceived growth in medical travel, whether through out-of-country care or medical tourism. To address this issue, we collaborated with the Canadian Medical Association (CMA) to conduct an on-line survey, described below. The CMA is a voluntary national professional association which represents 70% of the practicing medical profession in Canada.

## Methods

### Recruitment

Survey participants were recruited through the CMA member e-panel. This e-panel is a vehicle through which members voice their opinions on various topics through surveys administered electronically by the CMA. E-panel participants are informed that they will be anonymously surveyed maximally six times per year on different topics, and voluntarily give their informed consent through an established protocol.

### Sample

The sample consisted of physicians in Canada recruited through the CMA’s e-panel. The CMA’s e-panel is considered to fully represent the socio-demographic profile of CMA membership. Members include physicians at various points in their careers, from retired physicians to medical residents [7].

### Procedure

The *Cross border Health Care Survey* was sent to 3,169 CMA e-panel members on 29 September 2011. One follow-up reminder was sent to the sample population one week later.

### Survey questions

The *Cross border Health Care Survey*, which was issued in French and English (English version appended), consisted of seven main questions pertaining to the experiences and general perceptions of physicians regarding OOCC and MT. The questions were designed to give a general impression of the need for out-of-country treatment, number of referrals, approval rates and types of treatment, in addition to where patients had received OOCC or had been medical tourists. In order to gain knowledge of challenges related to both categories of care out-of-country, respondents were also invited to comment on physician responsibilities with regard to OOCC and MT patients, issues dealing with after-care upon patients’ return to Canada, information accompanying returning patients, and other issues. The survey contained both closed and open items.

### Analysis

Quantitative data were analyzed using descriptive statistics, including frequency distribution and cross-tabulations. Qualitative data were analyzed using qualitative description. Qualitative responses were compiled and categorized by common themes. One researcher initially coded the qualitative data with a second researcher reviewing the selection of categories.

## Results

631 of the 3,169 physicians in the e-panel completed the survey. This represented a response rate of approximately 20%, considered excellent by the CMA based on comparisons with past e-panel surveys. The results of the responses received are presented in both numeric and percentage forms where appropriate. Percentages with decimals have been rounded to the nearest whole percent. On occasion, respondents provided multiple answers to a question, contributing to a number of responses greater than the total number of respondents.

**Figure 1:**
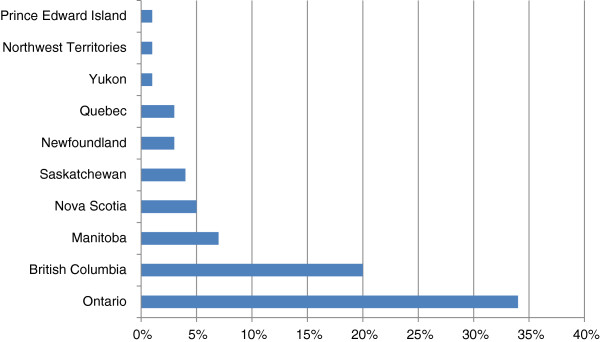
Respondents by location of practice.

Just over half (54%) of respondents were specialists;^a^ the rest were general practitioners. Figure 1 shows respondents by province of practice.

### Out-of-country care

28% of respondents (174) had determined that their patients required medically necessary treatment outside of Canada, to be paid for by a provincial/territorial health insurance plan; only 3% did not respond to the question, and the remainder indicated no such need. Those who responded in the affirmative were asked what percentage of these patients were given approval to have treatment outside Canada (Table 1), with just over a quarter indicating that all of their applications were given approval.

**Table 1:** **Percentage of respondents**’ **patients given approval for OOCC**

**Approval**	**Percent of patients**
Less than half	48%
Half	7%
More than half	4%
All	26%
Response errors	2%

**Figure 2:**
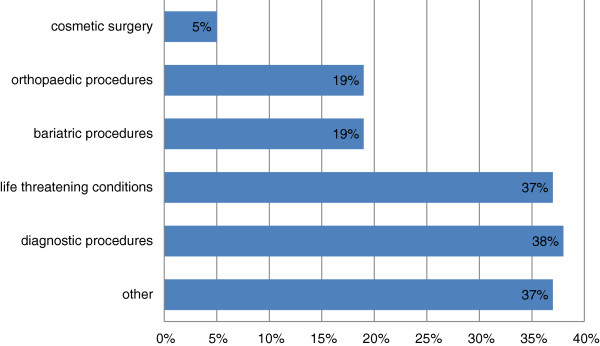
Types of OOCC treatments received.

167 respondents provided one or more answers to the sub-question “If yes, what types of treatment(s) did the patient receive outside Canada?” The majority of treatments received through out-of-country care involved diagnostic procedures, and a similar number for life threatening conditions (Figure 2).

Respondents were asked to identify the country where their OOCC patients had received treatment. Of the 159 responses received, 96% (152) indicated the United States. When combined, the other countries (Britain/UK, India, Mexico, Germany, Argentina, Belgium, Brazil, China, Estonia, France, Philippines, Poland and Switzerland and unspecified countries in Europe) accounted for very few responses.

### Medical tourism

Physicians were asked if they were aware if any of their patients had sought and independently paid for treatment outside Canada. Of the respondents, 66% (415/631) were aware of such cases, while 28% (177) of physicians responded no awareness. Just over 6% (39) did not record a response. Those who were aware of patients independently seeking treatment outside of Canada were asked about patients’ reasons for pursuing care (Table 2). Wait times, unavailability of treatment or inconvenient treatment locations were the three top-ranked reasons.

**Table 2:** R**easons for pursuing medical tourism**

**Reason for MT**	**Percent of responses**
Wait time in Canada too long	60% (257/415)
Procedure not available in Canada	33% (143/415)
Procedure not available in convenient location	9% (40/415)

Other reasons cited were related to the patients’ perception of better overall care, including expertise, and associated better attention, treatment and results, to be found abroad. For example, “(patients) think there is better expertise outside of Canada”, and “the patient falsely believed that U.S. was better,” and “perceived better quality in the U.S.” Another frequently cited ‘other’ reason was that patients sought second opinions regarding their condition and treatment, often from centres recognized for their excellence: “patient sought second opinion from ‘excellent centres’”; “second opinion at a prestigious medical center (Mayo)”, and “wanted a second opinion on diagnosis”.

**Figure 3:**
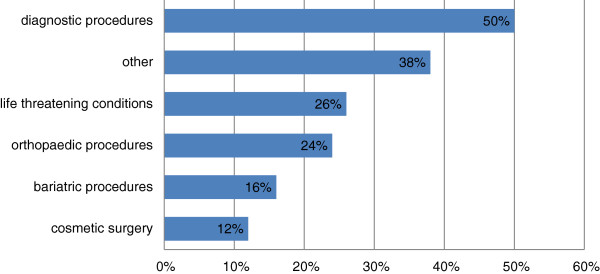
Types of treatments that patients independently purchased (MT).

Physicians who were aware of patients independently paying for treatment outside Canada were also asked to identify the types of treatments patients had sought. Similarly to the reasons for seeking out of country care, two of the first three sets of responses indicated diagnostic procedures, and treatment for life threatening diseases, although the second most mentioned category was ‘other’ (Figure 3).

While 38% (164) of responses were classified as “other”, many (49) indicated therapies related to multiple sclerosis (MS) (49), but also included a variety of non-specified surgical procedures, reproductive health care, ophthalmology and ‘routine’ cancer care, neurosurgery, mental health/addictions therapy, psychiatric care and others.

The main country where patients sought independently paid-for care was the United States with 85% (370/436) of the responses. Other countries made up the remaining 15% of the responses with Mexico, India, Poland, Costa Rica and China each having more than 10 responses.

### Complications and responsibility for providing follow-up treatment to medical tourists

**Figure 4:**
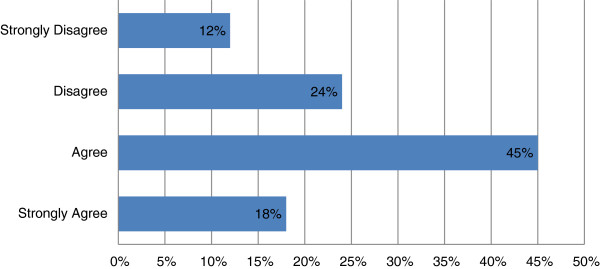
**Opinions on physician responsibility for follow**-**up treatment.**

Respondents were asked if they had had any issues dealing with health complications upon patients’ return, to which just over half (55%) indicated none, but 41% stated they had encountered such complications. Respondents were also asked if they felt it was their responsibility to provide follow-up treatment to medical tourists, with the majority agreeing (Figure 4) but a sizeable minority (36%) disagreeing. Those who disagreed about responsibility for providing follow-up treatment were twice as likely to report having had complications with patients on their return as those who agreed (35% vs. 17%). This suggests that physicians often see firsthand evidence of problems with medical tourism, and are raising questions about medical care that takes place outside the oversight and regulation of Canada’s health care system.

Respondents were quite clear about their responsibilities as physicians. As one respondent said, “It’s part of my job for goodness sake”, and another, “It is my duty to treat patients regardless of what treatment they received or did not receive”. However, some respondents expressed ambiguity towards their responsibility to provide care to medical tourists. For example, one respondent said “I only feel I have a responsibility to follow up if I have initiated the referral”. Another respondent expressed responsibility for a patient’s care only as long as that patient was not still seeking care at a foreign institution. If a patient was still going for treatment elsewhere, the respondent would defer to the source of their follow-up care (“barring medical emergency”). One respondent wrote “… it is my responsibility to follow up (ethically), but I do *not* necessarily agree that the publically funded system should pay for complications from these elective procedures” (emphasis in original).

### Issues related to information

Respondents who indicated that they had referred at least one patient outside Canada in the last five years for OOCC or were aware of their own patients travelling as medical tourists were asked if patients were returning to Canada with insufficient information about their treatment(s) abroad. 41% responded ‘yes’ to having insufficient information, while 55% responded ‘no’, and 4% indicated no response. Although not knowing if these respondents were the same as those who reported post-return complications, the exact congruence between these two items in response frequency suggests that lack of information and post-return complications may be related issues for Canadian physicians.

### Other issues

In addition to specific questions dealing with complications and insufficient information, respondents were asked in general if they had had any other issues. 70% of respondents to this question had no other issues to report, but 27% indicated that there were issues. The issue mentioned most often was related to communication. Comments included: “(there was) no translation of treatment” and “(issues related to) getting a hold of anyone in the treating clinic; getting information from the clinic; getting feedback about what treatment they initiated; what their plans for treatment were, etc”. Issues concerning practice abroad were also raised, for example, “sometimes service provided did not always seem clinically indicated. I have my own questions as to whether there is sometimes a financial incentive rather than clinical indication for prolonged length of stay”.

Others thought that patients expected too much of the Canadian health care system. For example, one respondent took issue with “patients demanding/asking for local follow-up treatment to be paid for by the hospital or provincial plan (e.g. physiotherapy)” (depending on the provincial public health plan, costs for ancillary services are typically borne out-of-pocket or through private, workplace-based insurance plans). Another noted “the individual’s attitude of entitlement” to ongoing monitoring and support, and another respondent said “the patient (was) requesting unreasonable amount of follow-up tests done here in Canada to be paid for publicly”.

## Discussion

The survey questions encouraged physicians to reflect on their experiences with MT and OOCC, and have provided some insights into physician concerns with medical travel. In summary, over a quarter of the sample of physician respondents had determined that one or more of their patients needed OOCC provided by provincial/territorial health insurance plans, and had applied for OOCC on their behalf. The rate of approval experienced by respondents was less than half for the 48% of those who responded, and fully successful in just over a quarter of the responses. The most common OOCC treatments received were diagnostic procedures followed closely by treatment for life threatening conditions, and the overwhelming majority of treatments were received in the United States.

Regardless of the procedure sought, respondents reported that their patients engaged in medical tourism because they considered waiting time for treatment in Canada was too long. An OECD report indicated that among 11 countries surveyed in 2010, Canada experienced some of the longest waiting times for health care; 59% of Canadians surveyed reported waiting four weeks or more to see a specialist, while 25% waited four months or more for elective surgery [5]. In 2012, nine out of ten Ontarians living in the eastern region of the province waited an average 343 days for hip replacement, making this the most poorly performing region in an already poorly performing province [8]. These wait times may not necessarily pose medical risks but, as one respondent in the physician survey wrote, patients experience “…anxiety re: waiting [even if] not founded on reality”.

The second most-reported reason for MT was because specific treatments were not available in Canada. In some provinces this applies to certain types of bariatric treatment which may only be available elsewhere in Canada than the patient’s home province, or outside the country. But bariatric care may also be supported through OOCC, as in Ontario. Demand for bariatric care through publicly-funded OOCC may have had some influence on recent decisions in Ontario to restructure bariatric services and increase domestic access [9].

An additional area of MT was for MS-related therapies. The respondents reported a large number of patients going abroad for such therapies, the majority mentioning procedures for Chronic Cerebrospinal Venous Insufficiency (CCSVI), also referred to in common parlance as liberation therapy or the Zamboni procedures. Due to lack of evidence of its efficacy, CCSVI treatment for MS is not currently funded by the public health care system in Canada [10]. In June 2011, the federal Minister of Health announced government funding for Phase I/II clinical trials on CCSVI [11] and by November, 2011, the government announced that it was ready to accept research proposals for these trials [12]. These steps suggest that public rather than physician demands had some influence on decisions to investigate care that is not available in Canada.

For both types of medical travellers (MT and OOCC), the top-ranked service sought abroad was diagnostic procedures which included CAT scans, MRIs and radiography. On the one hand, there is reason, as the literature and some of the respondents suggest, to believe these procedures may be overused [13-15]; on the other, prompt access in cases of medical necessity can allow patients to progress along the treatment spectrum. At the same time, the proximity of the United States, Canada’s border neighbour, which is well equipped to provide private diagnostic procedures, appears to be a critical factor in uptake.

Physicians’ perceptions regarding their responsibilities towards medical tourists, particularly their follow-up treatment are important to note. Though limited in number, there are some published studies indicating that returning patients can suffer significant problems sustained as a result of treatment abroad [16-20]. Our results suggest some differences of opinion within the e-panel regarding the provision of follow-up treatment to patients who have independently sought, paid for and received treatment outside the country. Several respondents were clearly uncomfortable with having to provide follow-up care for procedures performed by other practitioners, and for which they frequently lacked adequate information.

### Limitations

A potential limitation of the survey was that it was *the first enquiry into a new topic* conducted by the CMA. Despite piloting and refinement of the questions, it is possible that some physicians conflated medical tourism and out-of-country care. The survey was also limited to descriptive analyses. Although survey responses in general can help to prepare the ground for theoretical development, such as understanding the processes of physician-patient decision-making, theoretical development would require further enquiry. Another potential limitation of the survey is that the findings may not be generalizable to the experiences of the population of most physicians in Canada. Although the 20% response rate to this survey was impressive, it may be that those who decided to participate in this particular survey did so precisely because of their experiences with OOCC and MT. However, data collected including the types of treatment sought, the countries in which they were obtained, and the issues that Canadian physicians come across when dealing with patients’ OOCC and medical tourism, show likelihood of reflecting many physicians’ concerns and provide a good descriptive base upon which more evidence and subsequent policy considerations can be built.

## Conclusions

Out-of-country care and medical tourism are examples of how health care is becoming increasingly globalized. Both forms of cross-border care introduce some risks but may also generate efficiencies in health care delivery in Canada. Both risks and benefits, however, remain difficult to quantify given the paucity of hard data, especially with respect to MT. A sizeable number of physicians in our survey determined that their patients required OOCC and applied for public health insurance coverage (although approval was not guaranteed). By making requests for OOCC, physicians are responding to inadequacies that they perceive in the Canadian health care system. These inadequacies may well be most efficiently and cost-effectively filled by OOCC.

The main reasons for medical tourists seeking treatment outside Canada (to the best of respondents’ knowledge) were length of time waiting for treatment and treatment not being available in Canada. However better overall care, expertise, attention, treatment and results, and a desire for second opinions were also cited reasons for Canadians seeking care as medical tourists. These reasons reveal different kinds of issues with respect to the perceived quality or superiority of skills elsewhere. The availability of some novel therapies elsewhere but not in Canada, can also suggest that Canada is lagging behind many countries in terms of ‘state-of-the-art’ practice. However, medical tourism does appear to pose greater risks due to a greater number of unknowns for the patient including the security and safety of countries and the quality of care in their facilities, the regulation of practitioners and liabilities, the types of procedures and post-interventional follow-up or lack of it, and the effects of possible transmission of multiple drug, extremely drug and pandrug resistant bacteria/pathogens. For some of our respondents, MT raises worrying questions of who should bear responsibility and costs for complications arising from a personal decision to seek care in another country. Other studies have also raised concerns about the negative impacts that MT can bring about for health care access for population groups in some MT countries [21].

Hard data and further research in OOCC and MT could help identify the volume and impacts of these forms of medical travel on Canadian citizens and the Canadian health care system. Canadian physicians who witness through their patients the processes and experiences of OOCC and MT, would likely benefit from better support and information from health ministries, and streamlining of approval processes, but primarily from having access to timely services and treatment for their needy patients.

## Endnote

^a^Participants were not asked to indicate their area of specialization.

## Competing interests

The authors declare that they have no competing interests.

## Authors’ contributions

VR, CP, and RL were responsible for the study’s conception and initiation, data analysis and interpretation, and drafting and editing of the paper. SC participated in data analysis and interpretation and drafting of the paper. OA and JB contributed to the analysis and interpretation of the results, drafting and editing of the paper. All authors (with the exception of SC) participated in the survey design. All authors reviewed and approved the final submission.
